# A novel mechanism of immunity controls the onset of cinnamycin biosynthesis in *Streptomyces cinnamoneus* DSM 40646

**DOI:** 10.1007/s10295-016-1869-9

**Published:** 2016-11-17

**Authors:** Sean O’Rourke, David Widdick, Mervyn Bibb

**Affiliations:** Department of Molecular Microbiology, John Innes Centre, Norwich, UK

**Keywords:** Lantibiotic, Phosphatidylethanolamine, Regulation, Resistance, Induction

## Abstract

*Streptomyces cinnamoneus* DSM 40646 produces the Class II lantibiotic cinnamycin which possesses an unusual mechanism of action, binding to the membrane lipid phosphatidylethanolamine (PE) to elicit its antimicrobial activity. A comprehensive analysis of the cinnamycin biosynthetic gene cluster has unveiled a novel mechanism of immunity in which the producing organism methylates its entire complement of PE prior to the onset of cinnamycin production. Deletion of the PE methyl transferase gene *cinorf10*, or the two-component regulatory system (*cinKR*) that controls its expression, leads not only to sensitivity to the closely related lantibiotic duramycin, but also abolishes cinnamycin production, presumably reflecting a fail-safe mechanism that serves to ensure that biosynthesis does not occur until immunity has been established.

## Introduction

**Fig. 1:**
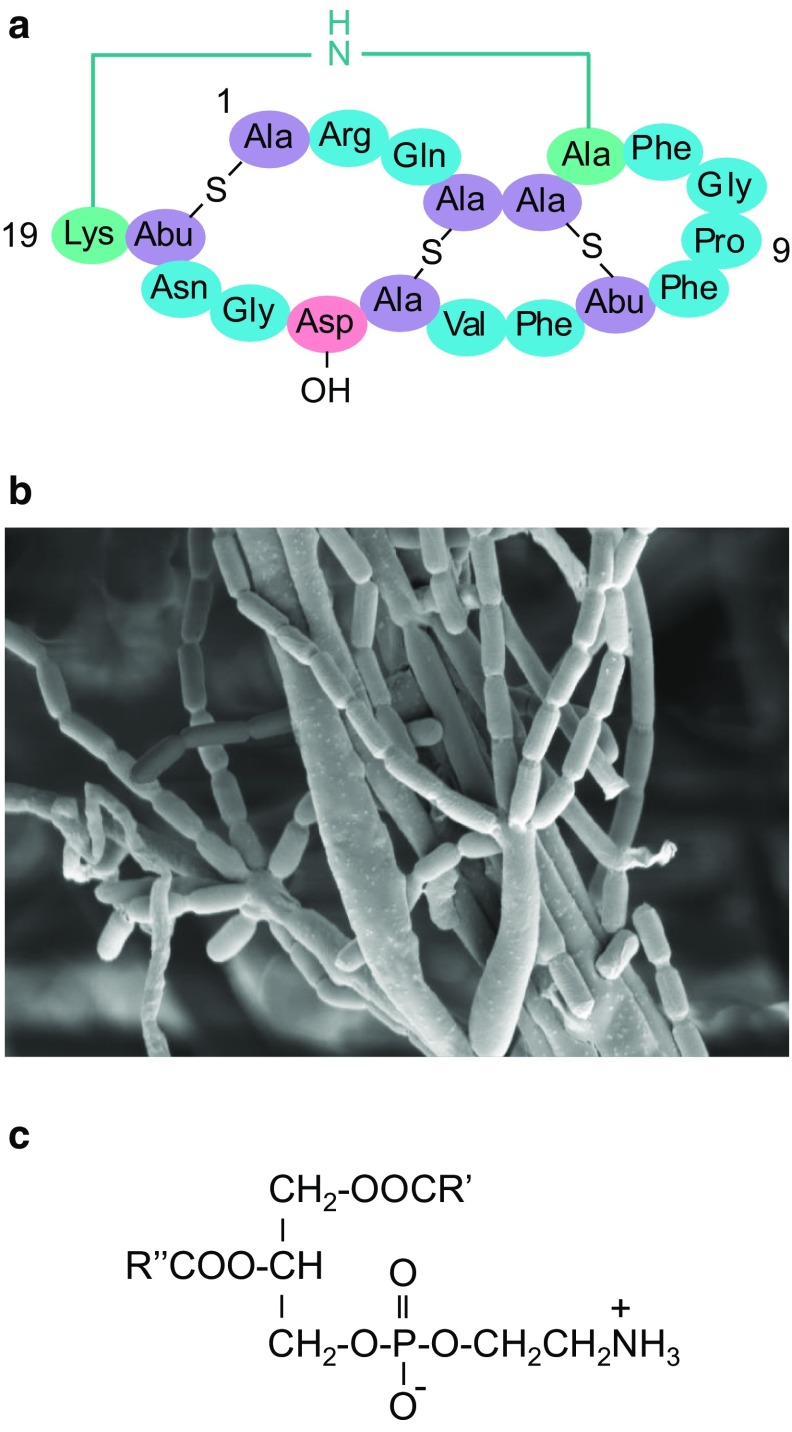
**a** Structure of cinnamycin with the unusual lysino-alanine bridge shown in *green*. **b** Scanning electron micrograph of *S. cinnamoneus*. **c** Structure of PE

Cinnamycin (Fig. 1a) is a Class II lantibiotic produced by *Streptomyces cinnamoneus* DSM 40646 (Fig. 1b) and belongs to the wider family of Ribosomally synthesised post-translationally modified Peptides (RiPPs) [2]. It is a member of a small group of related compounds that also include cinnamycin B, duramycin, duramycin B, and duramycin C made by other actinomycetes [12, 17, 20]. It is a potent antibiotic that is active against a broad range of Gram-positive bacteria and possesses an unusual mechanism of action, binding to the membrane amino phospholipid phosphatidylethanolamine (PE; Fig. 1c), a mechanism that is unique to the cinnamycin family of lantibiotics [10]. The ability of these compounds to bind to PE has led to the use of duramycin to investigate the function and localisation of the amino phospholipid in cell membranes, and to their potential application as diagnostic or therapeutic agents [15, 22]. PE is a major membrane lipid in streptomycetes [8], leading us to wonder how *S. cinnamoneus* protects itself from this potent compound at the onset of cinnamycin biosynthesis which commences upon entry into stationary phase in liquid culture. In earlier work, we cloned the cinnamycin biosynthetic gene cluster (*cin*) and expressed it heterologously in *Streptomyces lividans* [20]. Likely, functions were assigned to many of the genes in the cluster by bioinformatic analysis. Thus, *cinA* was proposed to encode the cinnamycin precursor peptide, *cinM* the lanthionine synthetase responsible for the introduction of the lanthionine and methyl-lanthionine bridges, *cinX* the hydroxylase required for modification of residue D15, *cinTH* an ABC transporter involved in the export of the cinnamycin, *cinKR* a two-component regulatory system of unknown function, and *cinR1* the activator of the putative operon encoding the cinnamycin biosynthetic machinery. Some of these functions have since been confirmed experimentally [14]. Here, we describe the results of a comprehensive analysis of the cinnamycin biosynthetic gene cluster in *S. cinnamoneus* that has unveiled a novel mechanism of immunity that exerts a controlling influence on whether cinnamycin production occurs or not.

## Mutational analysis of the *cin* gene cluster

**Fig. 2:**
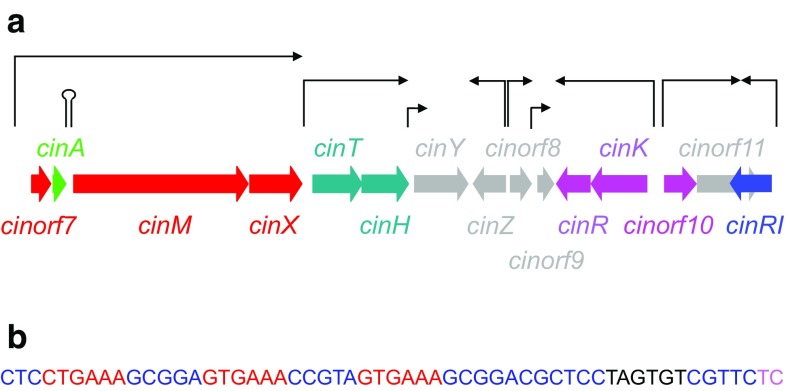
**a** Organisation of the *cin* cluster with transcriptional start sites identified by nuclease S1 protection studies shown by *black arrows* above the gene cluster together with the presumed extent of the resulting transcripts; the location of the transcriptional attenuator between *cinA* and *cinM* is shown by a hairpin loop. The gene (*cinA*) encoding the precursor peptide is shown in *light green*, genes (*cinorf7MX*) involved in its modification in *red*, those involved in immunity (*cinKRorf10*) in *pink*, those in transport (*cinTH*) in *dark green*, and the likely transcriptional activator of the *cinorf7AMX* operon, *cinR1*, in *blue*. **b** Promoter region of *cinorf7*. Nucleotides (TC) at the 5′ end of the likely *cinorf7AMX* transcript identified by nuclease S1 protection studies are shown in *pink*, the putative −10 RNA polymerase recognition sequence (TAGTGT) in *black*, and the three hexameric repeats in *red*

Previous work [20] had identified a cluster of 15 genes (Fig. 2a) that were potentially involved in cinnamycin biosynthesis. To assess their roles in production of the lantibiotic, null mutations were made in each of the presumptive protein coding sequences in *S. cinnamoneus* by PCR-targeting [7]. While individual deletion of *cinorf7AMXTHRKorf10R1* abolished cinnamycin production (determined by bioassays of culture supernatants using *Bacillus subtilis* EC1524 as indicator organism and confirmed by Matrix Assisted Laser Desorption Ionization Time-of-Flight (MALDI-ToF) mass spectrometry; data not shown), deletion of *cinX* resulted in the production of the inactive, non-hydroxylated form of cinnamycin (deoxycinnamycin), consistent with its role as an α-ketoglutarate/iron(II)-dependent hydroxylase [14]. Production of cinnamycin was restored in each of the non-producing mutants after introduction of the corresponding plasmid-borne wild-type gene. Deletion of *cinYZorf8orf9orf11* (for *cinorf11*, the segment not overlapping *cinR1* was deleted) had no effect on cinnamycin biosynthesis and the reason for their presence in the *cin* cluster is not known.

## Transcriptional organisation of the *cin* gene cluster and a key role for *cinR1* in activating cinnamycin production

To determine the transcriptional organisation of the *cin* gene cluster and to ultimately gain insights into the mechanisms underlying the regulation of cinnamycin production, nuclease S1 protection studies were carried out revealing at least nine transcriptional start sites (Fig. 2a), five of which are involved in expressing genes required for biosynthesis of the lantibiotic (organised in the likely the transcription units *cinorf7AMX*, *cinTH*, *cinKR*, *cinorf10,* and *cinR1*). *cinR1*, at the right end of the *cin* cluster, encodes a member of the SARP family of regulatory proteins [21]; the binding sites for these transcriptional activators are often characterised by a series of hexameric nucleotide repeats that are separated from each other by one turn (ca. 11 nt) of the DNA helix with the gene proximal repeat situated one and a half helical turns from the −10 promoter element recognised by RNA polymerase. Interestingly, three such repeats are located upstream of the *cinorf7* transcriptional start site (Fig. 2b). Together with the 1800-fold reduction (determined by qRTPCR; data not shown) in the level of transcription of *cinorf7* upon deletion of *cinR1*, this suggests that CinR1 is the direct transcriptional activator of the likely *cinorf7AMX* biosynthetic operon. Previously, we had noted the presence of an inverted repeat sequence located between *cinA* and *cinM* [20]; nuclease S1 protection studies revealed that this sequence, as we had speculated, functions as a transcriptional attenuator, presumably ensuring an appropriate stoichiometry of the precursor peptide CinA and the biosynthetic enzymes CinM (lanthionine synthetase) and CinX. While Cinorf7 has been shown to be required for formation of the lysino-alanine bridge [14], no enzymatic function for the protein has been identified; given that it is likely to be produced in similar quantities to the precursor peptide, it is conceivable that it performs a chaperone-like role in the formation of this unusual post-translational modification.

## *cinorf10* encodes a PE monomethyltransferase

**Fig. 3:**
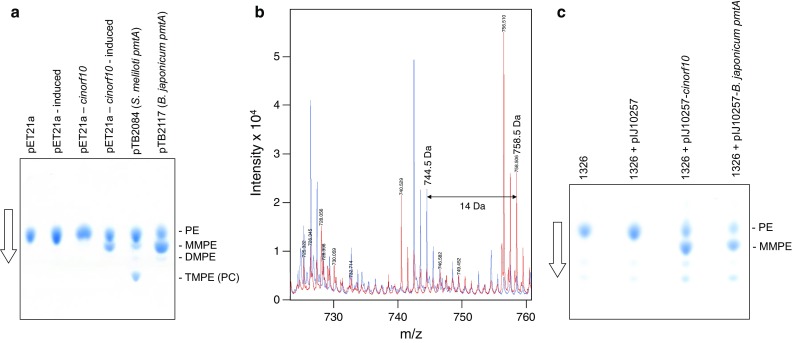
*cinorf10* encodes PEMT activity. **a** *cinorf10* coding sequence was amplified by PCR from *S. cinnamoneus* genomic DNA, cloned into the isopropyl β-d-1-thiogalactopyranoside (IPTG)-inducible expression vector pET21a, and introduced into *E. coli* BL21 (DE3) by transformation. Duplicate cultures were grown in LB liquid medium and one induced by the addition of 0.4 mM ITPG; lipids were extracted 2 h later and subjected to one-dimensional TLC with (propan-1-ol)-propionic acid-chloroform-water (3:2:2:1) and the plates developed with Dittmer–Lester Reagent. Additional controls included *E. coli* cultures expressing the *pmtA* genes from *S. meliloti* or *B. japonicum*. The *arrow* indicates the direction of chromatography. **b** In *E. coli*, PE consists of a mixture of molecules with different acyl chains [1]. The figure shows super-imposed segments of the [M + H]^+^ mass spectra obtained with lipids isolated from *E. coli* BL21 (DE3) containing either pET21a (*blue*) or pET21a-*cinorf10* (*red*) after induction with IPTG. Note the efficient conversion of the presumed PE C36:2 (*m*/*z* [M + H]^+^ = 744.5), which is a recognised species in *E. coli* [16], to the likely methylated form with a mass of 758.5 Da. The identities of the peaks at *m*/*z* [M + H]^+^ = 742.5 and [M + H]^+^ = 756.5 Da do not correspond to previously identified PE species (note that the height of the peak with *m*/*z* [M + H]^+^ = 744.5 Da is slightly larger than the second isotope peak of the *m*/*z* [M + H]^+^ = 724.5 species indicating that it is a distinct entity). **c** *cinorf10* and *B. japonicum pmtA* were cloned downstream of the constitutive ermE* promoter in the integrative vector pIJ10257 in *E. coli* and the resulting plasmids, as well as the vector control, introduced into *S. lividans* 1326 by conjugation. The cultures were grown for 24 h in TSB liquid medium, lipids extracted, and subjected to TLC analysis as described in **a**

BlastP analysis of Cinorf10 suggested that it might possess PE methyltransferase (PEMT) activity. To address this possibility, the *cinorf10* coding sequence was amplified by PCR from *S. cinnamoneus* genomic DNA, cloned into the pET21a expression vector (Novagen), and introduced into *Escherichia coli* BL21 (DE3). Thin layer chromatography (TLC) analysis of lipid extracts from the induced cells revealed an additional spot with a similar mobility to monomethylphosphatidylethanolamine (MMPE) observed in lipid extracts of *E. coli* cells expressing the functionally characterised *pmtA* genes of either *Bradyrhizobium japonicum* [13] or *Sinorhizobium meliloti* [3] (Fig. 3a). The bradyrhizobial *pmtA* produces predominantly MMPE and a smaller amount of dimethyl phosphatidylethanolamine (DMPE) when expressed in *E. coli*, while cells expressing the sinorhizobial *pmtA* accumulate predominantly trimethyl phosphatidylethanolamine (TMPE), more commonly known as phosphatidylcholine (PC), although some mono- and dimethylated phosphatidylethanolamine could also be detected. Further evidence that the novel lipid spot observed in *E. coli* cultures expressing *cinorf10* did, indeed, contain MMPE was obtained by mass spectrometry (Fig. 3b).

## Expression of *cinorf10* in *S. lividans* results in MMPE accumulation and confers resistance to duramycin

The *cinorf10* coding sequence was next cloned into the integrative *Streptomyces* vector pIJ10257 [9] under the control of the constitutive ermE* promoter and introduced into *S. lividans* 1326 by conjugation. TLC analysis of lipid extracts of the *cinorf10*-containing strain again revealed an additional lipid spot, the mobility of which was again consistent with MMPE (Fig. 3c). This additional lipid spot was not detected in a strain containing only the pIJ10257 vector, but a spot of similar mobility was observed in extracts from the strain expressing the bradyrhizobial *pmtA*.

**Fig. 4:**
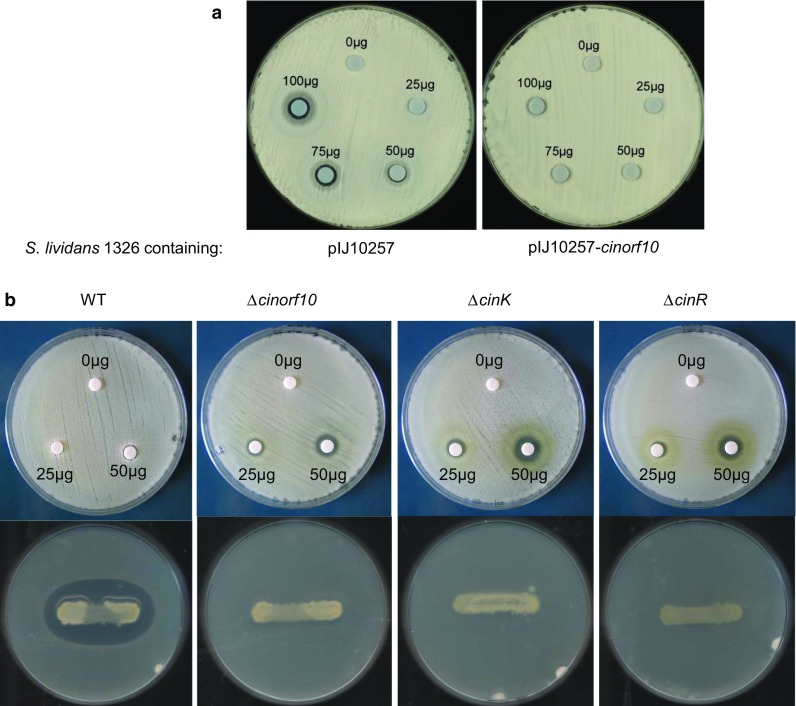
**a** Expression of *cinorf10* in *S. lividans* confers immunity to duramycin. Confluent lawns of spores of *S. lividans* 1326 and the strain containing pIJ10257-*cinorf10* were spread onto R2 agar plates, and assay disks containing varying amounts of duramycin were applied to the plate. **b** *Top* Deletion of *cinorf10, cinK,* or *cinR* in *S. cinnamoneus* results in sensitivity to duramycin. See **a** for assay details. *Bottom* Deletion of *cinorf10, cinK,* or *cinR* in *S. cinnamoneus* results in loss of cinnamycin production. Streaks of each of the *S. cinnamoneus* strains were overlaid with soft nutrient agar containing *B. subtilis* as indicator

To determine if the accumulation of MMPE could confer resistance to cinnamycin in *S. lividans*, the strain expressing *cinorf10* was challenged with the closely related commercially available duramycin. Duramycin differs from cinnamycin by a single amino acid substitution (R2K), but shares an identical mode of action [11]. Expression of *cinorf10* in the heterologous host appeared to confer complete resistance to the lantibiotic (Fig. 4a).

## Deletion of *cinL*, *cinR,* or *cinK* in *S. cinnamoneus* results in sensitivity to duramycin as well as abolishing cinnamycin production

The mutational analysis described earlier had shown that deletion of *cinorf10*, *cinR,* or *cinK* in *S. cinnamoneus* resulted in loss of cinnamycin production. To assess the effect of these deletions on immunity to cinnamycin, bioassays were carried out revealing that each of the mutants had become sensitive to duramycin (Fig. 4b), consistent with a role for *cinKR* in regulating *cinorf10* expression and potentially that establishment of immunity is a pre-requisite for biosynthesis. Both resistance to duramycin and notably cinnamycin production was restored to each of the deletion strains following the introduction of pIJ10257 with *cinorf10* expressed from the ermE* promoter (data not shown); the ability of constitutively expressed *cinorf10* to suppress the non-producing phenotype of the mutants indicates that the only essential role for CinKR in cinnamycin biosynthesis is to regulate *cinorf10* expression.

## Expression of PEMT activity is induced by sub-inhibitory concentrations of duramycin

**Fig. 5:**
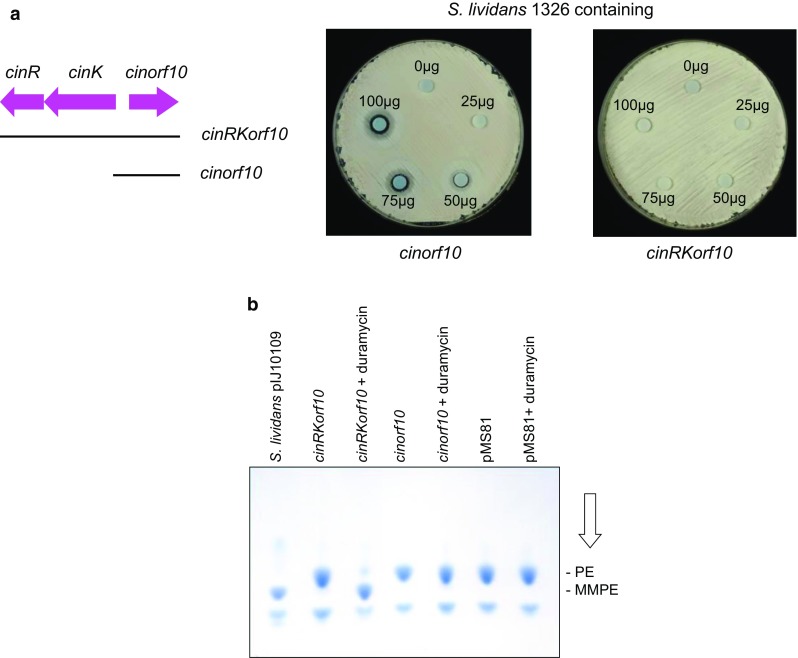
**a** *cinKR* are required for expression of *cinorf10* from its own promoter in *S. lividans*. The segment of the *cin* cluster cloned in pMS81 is shown below the gene map. Bioassays were carried out as in Fig. 4a. **b** PEMT activity is dependent on *cinKR* and requires induction with duramycin. The *S. lividans* derivatives described in **a**, together with the vector (pMS81) control, were grown in duplicate in TSB liquid medium; after 8 h, a sub-inhibitory concentration (1 µg/ml) of duramycin was added to one of the duplicates and all cultures grown for an additional 16 h. *S. lividans* containing pIJ10109 (carrying the entre *cin* cluster) was grown in TSB for 24 h as a further control. Lipids were then extracted and subjected to TLC analysis

To confirm the roles of *cinR*, *cinK,* and *cinorf10* in MMPE accumulation and duramycin resistance, segments of the *cin* cluster containing all three genes or just *cinorf10* (with its own promoter) were introduced into *S. lividans* 1326 using the integrative vector pMS81 [6]. The ability of the plasmids to confer resistance to duramycin and to promote the formation of MMPE was then assessed. While the strain containing *cinorf10* remained sensitive to duramycin, the derivative containing all three genes appeared completely resistant (Fig. 5a), consistent with a role for *cinKR* in regulating the expression of *cinorf10*.

**Fig. 6:**
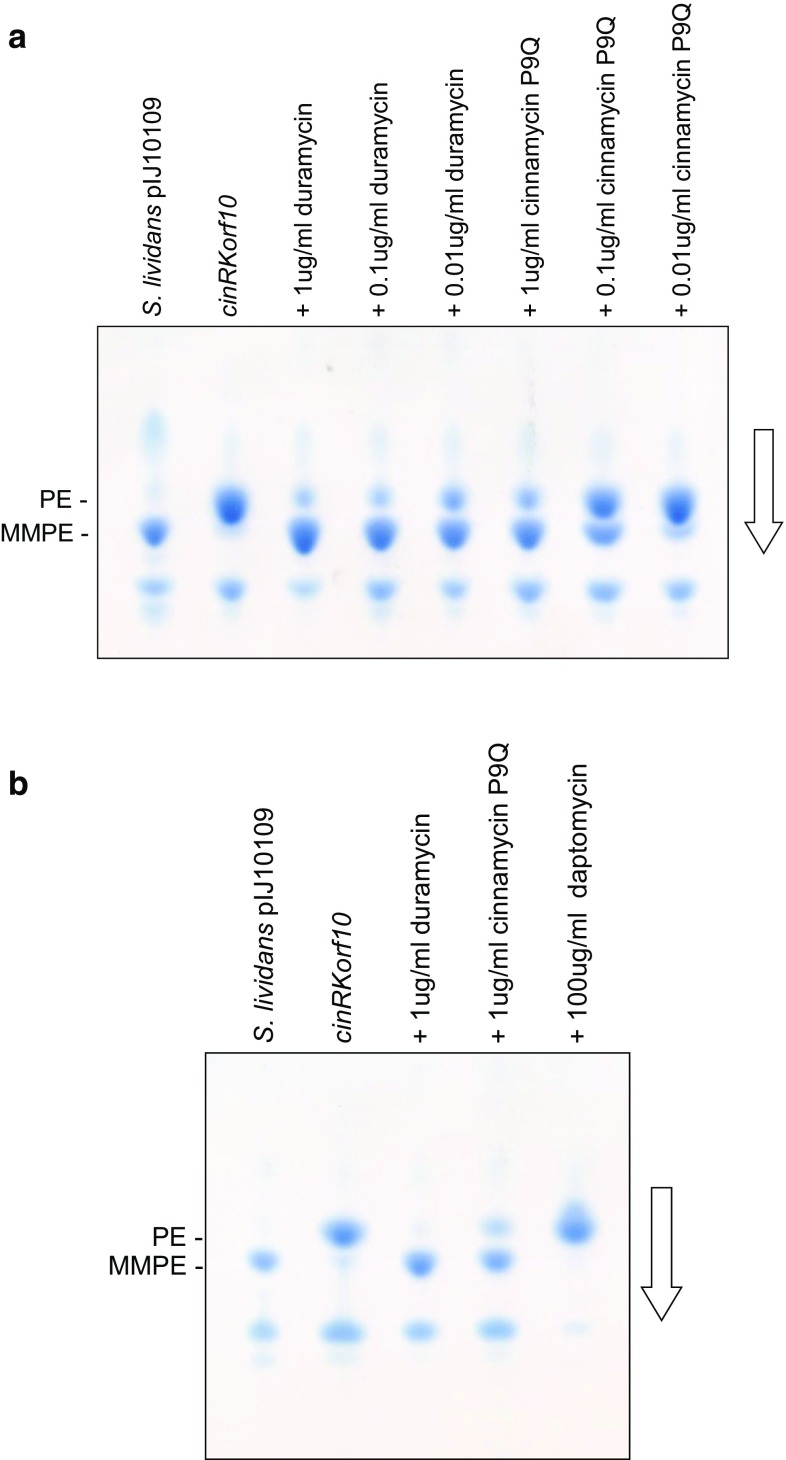
**a** PEMT activity can be induced with inactive cinnamycin P9Q. *S. lividans* 1326 cultures containing *cinKRorf10* cloned in the integrative pMS81 vector were grown in TSB liquid medium for 24 h after addition of various amounts of duramycin or the inactive cinnamycin variant P9Q at 8 h. Lipids were then extracted and subjected to TLC analysis. **b** PEMT activity is not induced by membrane active daptomycin. Quadruplicate cultures of the same strain were grown in TSB liquid medium; after 8 h, a sub-inhibitory concentration of duramycin was added to one culture, the inactive cinnamycin P9Q to another, and daptomycin at a concentration that resulted in a fourfold reduction in growth rate and biomass accumulation to a third; nothing was added to the fourth. The cultures were grown for a total of 24 h, harvested, and lipids analysed by TLC. In both **a** and **b**, *S. lividans* containing pIJ10109 was used as a control for chromatography

Analysis of lipid extracts of the duramycin resistant strains grown in liquid culture in the absence of the lantibiotic failed to reveal the presence of MMPE, although PE could be readily detected (Fig. 5b). However, addition of sub-inhibitory concentrations of duramycin to the cultures led to MMPE accumulation consistent with an inducible immunity mechanism. Comparable levels of induction were observed over a wide range (0.01–1 µg/ml) of sub-inhibitory concentrations of duramycin (Fig. 6a).

In an attempt to address whether induction might reflect a direct interaction of duramycin with CinK, rather than membrane stress sensed by the sensor kinase, cultures were also treated with a variant of cinnamycin (P9Q) that lacks antimicrobial activity and that is thought not to bind to PE. In cinnamycin, P9 contributes to the PE-binding pocket and may interact directly with the ethanolamine head group of the phospholipid [10, 19]. Replacement of P9 with a Q (glutamine) residue containing a bulky side group would likely interfere with the insertion of the glycerophosphoethanolamine head group into its binding pocket. The ability of the variant to induce PEMT activity (Fig. 6a) suggests that induction is not a consequence of membrane stress. Moreover, since immunity to cinnamycin appears to be a pre-requisite for production, the ability of the strain to produce deoxycinnamycin, which lacks antibiotic activity, after deletion of *cinX* is again consistent with specific induction by the lantibiotic, rather than a response to membrane perturbation.

When cultures were treated with daptomycin, a cyclic lipopeptide antibiotic produced by *Streptomyces roseosporus* and whose lipophilic tail is believed to insert into the bacterial cell membrane causing rapid membrane depolarization and potassium ion efflux, and thus membrane stress [18], at concentrations that caused a fourfold reduction in growth rate and biomass accumulation, there was no induction of PEMT activity (Fig. 6b), again consistent with a direct role for the lantibiotic independent of its antimicrobial activity in inducing immunity.

## Expression of PEMT activity occurs in a growth phase-dependent manner and precedes cinnamycin production

**Fig. 7:**
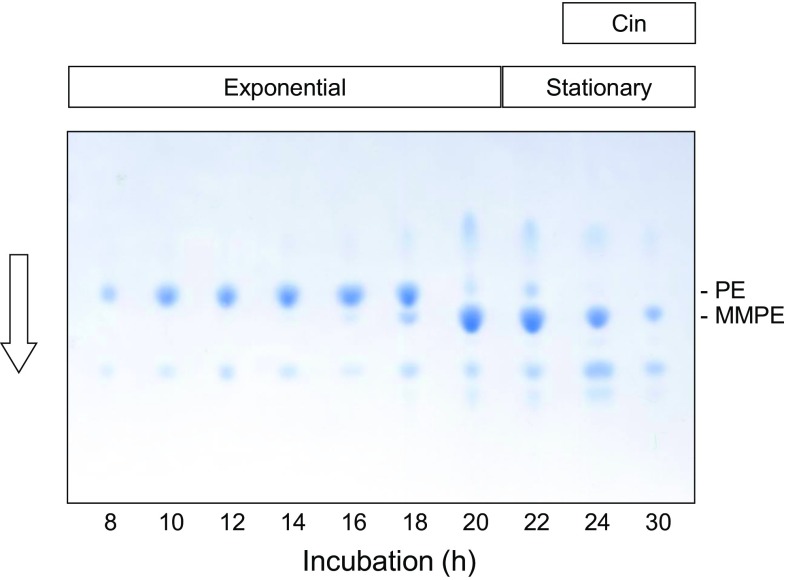
Growth phase-dependent methylation of PE in *S. lividans* containing the cloned *cin* cluster. TLC analysis of extracted membrane lipids of *S. lividans* 1326 containing pIJ10109. Exponential and stationary phases of growth are indicated, together with the appearance of cinnamycin in culture supernatants (determined by MALDI-ToF mass spectrometry)

**Fig. 8:**
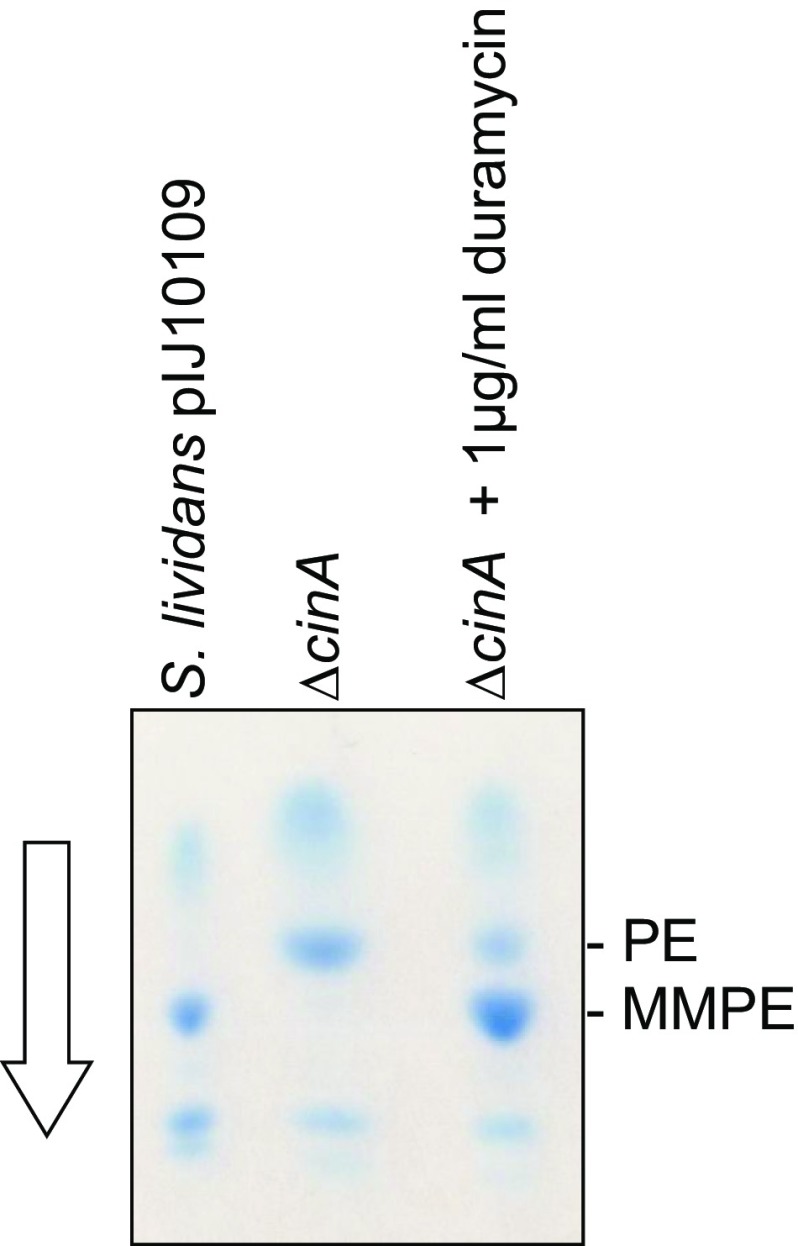
*cinA* is required for PEMT activity. *S. lividans* 1326 containing pIJ10109 and duplicate cultures of a derivative from which *cinA* had been deleted (in-frame deletion) were grown in TSB liquid medium. Duramycin (1 µg/ml) was added to one of the Δ*cinA* cultures after 8 h of incubation and all of the cultures harvested after a total of 24 h. Lipids were extracted and analysed by TLC

The results described thus far suggest that expression of *cinorf10* and establishment of immunity to cinnamycin might be a pre-requisite for cinnamycin production. To investigate this relationship further, *S. lividans* containing the entire *cin* cluster present in pIJ10109 [20] was grown in liquid culture, and the methylation status of PE and cinnamycin production monitored throughout growth (Fig. 7). MMPE was first apparent in late exponential growth phase (around 16 h after inoculation) and increased in abundance relative to PE during transition phase (20 and 22 h). By 24 h (stationary phase), all of the PE appeared to be in the methylated form, and it was at this point that cinnamycin could first be detected in the culture supernatant by MALDI-ToF mass spectrometry. We suggested above that cinnamycin itself might induce expression of *cinorf10*, and that establishment of immunity was necessary for biosynthesis to occur. Consistent with this hypothesis, deletion of *cinA* from the heterologous gene cluster in *S. lividans* resulted in loss of PEMT activity as well as the expected abolition of cinnamycin production; PEMT activity was restored by the exogenous addition of sub-inhibitory concentrations of duramycin (Fig. 8).

## Deletion of *cinorf10* abolishes cinnamycin production but has no effect on *cinorf7* and *cinM* transcription

**Fig. 9:**
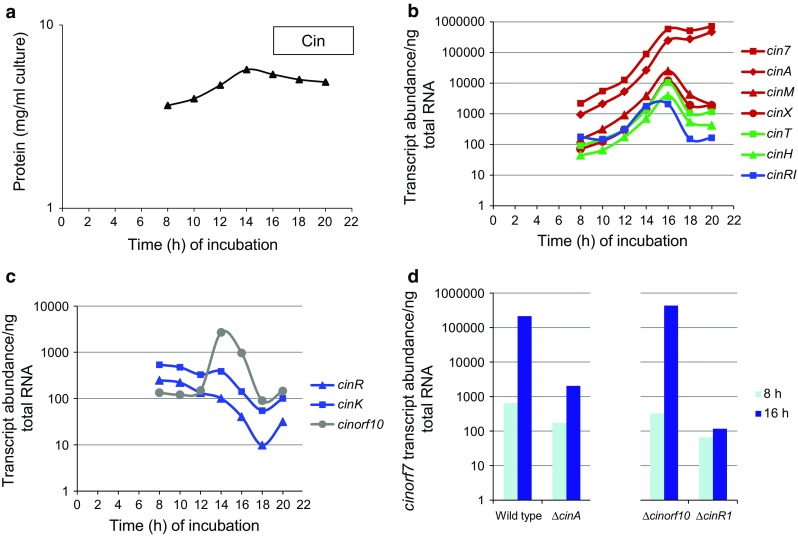
qRTPCR analysis of *cin* gene expression in *S. cinnamoneus*. **a** Growth curve of *S. cinnamoneus* with biomass estimated by measuring total protein content. **b** qRTPCR analysis of *cinorf7AMXTHR1* transcription during growth. **c** qRTPCR analysis of *cinKRorf10* transcription during growth. **d** qRTPCR analysis of *cinorf7* transcription after 8 h and 16 h of incubation in the wild-type strain and individual *cinAorf10R1* mutants. The results shown are the averages obtained from triplicate cultures

Since deletion of *cinorf10* abolished cinnamycin production in *S. cinnamoneus*, we set out to assess the effect of the mutation on transcription of *cinorf7* and thus the *cinorf7AMX* operon. The wild-type strain was first cultured in TSB liquid medium, RNA isolated at different times during growth and subjected to qRTPCR (real-time quantitative reverse transcription PCR) analysis (Fig. 9). Cinnamycin production was first detected (by MALDI-ToF mass spectrometry) in stationary phase after 16 h of incubation (Fig. 9a), corresponding to maximal levels of transcript abundance of *cinMXTHR1* which subsequently declined (Fig. 9b). The levels of the *cinorf7A* transcripts, which were markedly higher than those of *cinMX*, presumably reflecting the presence of the transcriptional attenuator located between *cinA* and *cinM*, remained close to their maximal levels beyond 16 h of incubation. Transcription of *cinKR* did not increase upon entry into stationary phase, but declined somewhat, consistent with a role in sensing exogenous cinnamycin. Interestingly, transcript levels for *cinorf10* peaked after 14 h, before those of the other *cin* genes, and before cinnamycin could be detected in the culture supernatant, again consistent with a role in establishing immunity to cinnamycin prior to the onset of detectable production. We next assessed the effect of mutations in *cinAorf10R1* on *cinorf7* and *cinM* transcription in RNA samples isolated after 8 and 16 h of incubation (Fig. 9d; since the data acquired for *cinorf7* and *cinM* are essentially the same, consistent with the existence of a *cinorf7AMX* operon, only the results for *cinorf7* are presented). Activation of transcription of *cinorf7* and *cinM* was essentially abolished in the *cinR1* mutant, consistent with the latter’s proposed role as the direct transcriptional activator of the *cinorf7AMX* operon. Deletion of *cinA* caused a marked reduction in the levels of induction, but did not completely abolish it, commensurate with a role for cinnamycin in promoting maximal levels of *cinorf7AMX* transcription. Surprisingly, since the mutant fails to produce the lantibiotic, deletion of *cinorf10* had no effect on *cinorf7* or *cinM* transcription.

## The lysino-alanine bridge of cinnamycin is required for antibacterial activity

Our earlier deletion analysis had shown that *cinorf7* is essential for cinnamycin biosynthesis in *S. cinnamoneus*. More recently, co-expression studies in *E. coli* have shown that the same gene was required for lysino-alanine bridge formation and that the latter was required for antibacterial activity [14]. In a complementary approach, the substrate flexibility of CinM and CinX was exploited using an in vivo expression system developed by Jesus Cortes (Novacta Biosystems) to generate cinnamycin variants in *S. cinnamoneus* in which S6 or K19 were replaced with A. Production of both variants (S6A, 2042 Da; K19A, 1983 Da) was readily confirmed by MALDI-ToF mass spectrometry, but both failed to inhibit *B. subtilis* in bioassays, confirming the previous *E. coli* expression studies.

## Conclusions

**Fig. 10:**
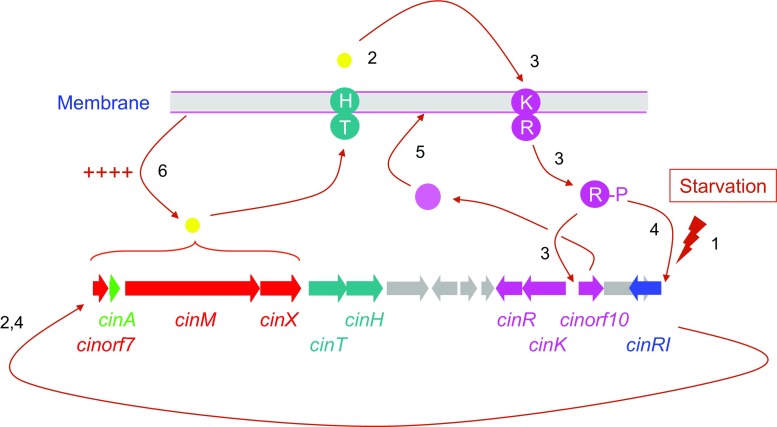
Model for regulation of cinnamycin production. Steps in activating expression of the *cin* cluster are numbered in the order in which they are proposed to occur

The results described here allow us to present a model for the regulation of cinnamycin biosynthesis (Fig. 10). We propose that activation of expression of the *cin* cluster is initiated by nutrient limitation that results in a low level of transcription of *cinR1* (1). This results in a low level of expression of the proposed *cinorf7AMX* operon and the production of a small amount of cinnamycin that is exported from the cell by CinTH (2). Cinnamycin then interacts with the membrane-associated sensor kinase CinK which results in phosphorylation of CinR and activation of transcription of *cinorf10* encoding the PEMT (3). CinR-P may also further activate transcription of *cinR1* (transcription of *cinR1* in a *cinR* mutant is reduced tenfold compared with the wild-type strain, data not shown) (4), resulting in higher levels of transcription of the *cinorf7AMX* operon (4). In the meantime, Cinorf10 proceeds to methylate PE in the membrane (5) and only when all of the PE has been modified does high-level production of cinnamycin occur (6). Intriguingly, while deletion of *cinorf10* abolishes cinnamycin production, it appears to have no effect on the level of transcription of the *cinorf7AMX* operon, implying the existence of post-transcriptional regulatory feedback mechanism that functions to ensure that cinnamycin production only occurs once immunity to the lantibiotic has been established. The existence of an apparent fail-safe mechanism such as this that presumably serves to prevent the strain from deleterious antibiotic production before the establishment of immunity was also observed in microbisporicin biosynthesis in *Microbispora corallina*, where deletion of an apparent immunity transporter also abolished lantibiotic production [4, 5]. In the latter case, microbisporicin was shown to be able to induce its own synthesis; it will be interesting to determine whether cinnamycin possesses the same property, or alternatively that the immunity mechanism that we have described functions only to protect *S. cinnamoneus* from cinnamycin produced either by itself or other members of the microbial community in which it exists in nature.
